# Effects of Anthocyanins on CAG Repeat Instability and Behaviour in Huntington’s Disease R6/1 Mice

**DOI:** 10.1371/currents.hd.58d04209ab6d5de0844db7ef5628ff67

**Published:** 2016-07-05

**Authors:** Linda Møllersen, Olve Moldestad, Alexander D. Rowe, Anja Bjølgerud, Ingunn Holm, Linda Tveterås, Arne Klungland, Lars Retterstøl

**Affiliations:** Institute of Medical Microbiology, Oslo University Hospital, Rikshospitalet, Oslo, Norway; Centre for Rare Disorders, Oslo University Hospital, Rikshospitalet, Oslo, Norway; Institute of Medical Microbiology, Oslo University Hospital, Rikshospitalet, Oslo, Norway; Institute of Medical Microbiology, Oslo University Hospital, Rikshospitalet, Oslo, Norway; Department of Medical Genetics, Oslo University Hospital, Ullevål, Oslo, Norway; Institute of Medical Microbiology, Oslo University Hospital, Rikshospitalet, Oslo, Norway; Institute of Medical Microbiology, Oslo University Hospital, Rikshospitalet, Oslo, Norway; Department of Medical Genetics, Oslo University Hospital, Ullevål, Oslo, Norway

## Abstract

Background: Huntington’s disease (HD) is a progressive neurodegenerative disorder caused by CAG repeat expansions in the *HTT* gene. Somatic repeat expansion in the R6/1 mouse model of HD depends on mismatch repair and is worsened by base excision repair initiated by the 7,8-dihydroxy-8-oxoguanine-DNA glycosylase (Ogg1) or Nei-like 1 (Neil1). Ogg1 and Neil1 repairs common oxidative lesions.

Methods: We investigated whether anthocyanin antioxidants added daily to the drinking water could affect CAG repeat instability in several organs and behaviour in R6/1 HD mice. In addition, anthocyanin-treated and untreated R6/1 HD mice at 22 weeks of age were tested in the open field test and on the rotarod.

Results: Anthocyanin-treated R6/1 HD mice showed reduced instability index in the ears and in the cortex compared to untreated R6/1 mice, and no difference in liver and kidney. There were no significant differences in any of the parameters tested in the behavioural tests among anthocyanin-treated and untreated R6/1 HD mice.

Conclusions: Our results indicate that continuous anthocyanin-treatment may have modest effects on CAG repeat instability in the ears and the cortex of R6/1 mice. More studies are required to investigate if anthocyanin-treatment could affect behaviour earlier in the disease course.

## Introduction

Huntington’s disease (HD) is a progressive neurodegenerative disorder caused by a CAG expansion in exon 1 of the *Huntingtin* *(HTT)* gene encoding the polyglutamine protein HTT 1. There is an inverse relationship between CAG repeat length and age of onset 2. Mouse Htt is ubiquitously expressed and the function of the normal protein is still under extensive investigation, although it is known to interact with trafficking motors and clathrin-interacting protein 3. A selective pattern of neuropathology exist in HD, with loss of neurons that is most severe in the caudate and putamen 1. However, the mechanisms underlying this selective neurodegeneration remain poorly understood. Somatic CAG length expansion is correlated with neuropathology and probably precedes the onset of symptoms 4. The R6/1 transgenic mouse is a widely used HD model containing the human *HTT* N-terminal fragment containing exon 1 with expanded CAG repeats 5. We have recently shown that the striatum and the cortex in R6/1 mice display a dramatic and periodic expansion that is mechanistically different from the slow expansion observed in most other somatic tissues 6. Stoichiometries of base excision repair (BER) proteins correlates with the degree of somatic instability seen in the striatum and cerebellum of HD transgenic mice 7. Similarly, age-dependent CAG repeat expansion are reduced in R6/1 mice lacking the BER enzyme 7,8-dihydroxy-8-oxoguanine (8-oxoG)-DNA glycosylase (Ogg1) 8. Also, the BER DNA glycosylase Nei-like 1 (Neil1) has recently been shown to be a genetic modifier of both somatic and germline CAG repeat instability in R6/1 mice 9. Deletion of the mismatch repair proteins Msh2 and Msh3 has been shown to abolish somatic expansion in several HD mouse models 10 ^,^ 11 ^,^ 12. Recently, the mismatch repair genes *Mlh1* and *Mlh3* have also been shown to modify CAG instability in HD mice 13.

It has been proposed that oxidative damage plays a role in the progression of several neurodegenerative diseases 14. Reactive oxygen species are generated as by-products of mitochondrially catalysed reactions of the electron transport chain or cellular inflammation. Mice with expanded polyglutamine have shown Htt on neuronal mitochondrial membranes 15 with elevated levels of 7,8-dihydroxy-8-oxoguanine and lipid peroxidation 16, and mitochondrial dysfunction 17. It has been shown that the presence of aggregated mutant Htt fragments directly causes free radical production 18.

Several anthocyanins and their aglycones have shown strong antioxidant activity 14 ^,^ 19. Anthocyans are flavonols, which occur ubiquitously in the plant kingdom and confer bright red or blue colouration on berries and other fruits and vegetables. Blackcurrant *(Ribes* *nigrum)* and bilberry *(Vaccinium* *myrtillus)* - which is very similar to blueberry *(Vaccinium* *corymbosum)* - are rich in several anthocyanins. Anthocyanins can be metabolised in the intestine and enter the bloodstream to peripheral tissues (reviewed by 20 ^,^ 21). Anthocyanins from dietary blueberry has been detected in several brain regions, including cerebellum, cortex, hippocampus and striatum of rats 22 ^,^ 23, and cerebellum, cortex, midbrain and diencephalon in pigs 24. Blueberry anthocyanins have been shown to enhance cognitive and motor behaviour in aged rodents 22 ^,^ 23 ^,^ 25 ^,^ 26 ^,^ 27 ^,^ 28 ^,^ 29. Wild blueberry juice also improved paired associate learning and word list recall in older human adults with early memory changes 30. Blueberry extract also reduced oxidative DNA damage in mouse brain tissue *in vitro* as evaluated by the comet assay 26.

At present, there are no curative therapies available for HD. R6/1 HD mice have reduced motor coordination and cognitive deficits 31 ^,^ 32 ^,^ 33.Several antioxidants have been tested on animal models of HD, for instance a combination of coenzyme Q10 and remicade hydrochloride 34, alpha-lipoic acid 35, BN82451 36, resveratrol 37, fisetin 38, and *N*-Acetylcysteine 39, and have shown varying effects on survival, weight loss and rotarod performance.

We investigated whether anthocyanin antioxidants could reduce CAG repeat expansion and improve behavioural performance in the R6/1 mice. R6/1 HD mice were given Medox^®^, containing a combination of anthocyanins derived from bilberry and blackcurrant, in their drinking water from 4 weeks of age until they were sacrificed at 22 weeks of age. Several organs including male gonads and brain tissues were harvested to examine whether anthocyanins could reduce CAG repeat expansion in R6/1 mice. Before termination of the experiment, the exploratory behaviour of the mice was investigated in the open field, and balance and coordination was tested on an accelerating rod (rotarod test).

## Results

**The effect of anthocyanins on body weight of R6/1 HD mice**

All mice were weighed regularly to check their health throughout the experiment. The growth curves for female and male anthocyanin-treated and untreated R6/1 HD mice are shown in Fig. 1. The growth rate of female and male R6/1 HD mice is indistinguishable between anthocyanin-treated and untreated mice up to 10 weeks of age.

**Growth curves for anthocyanin-treated and untreated R6/1 HD mice.**
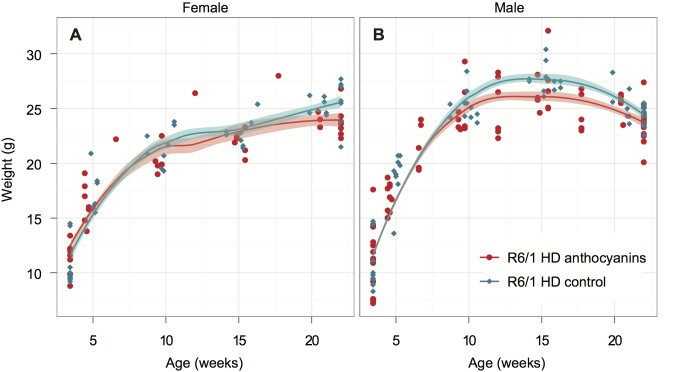
The R6/1 HD mice were grouped by gender and shown with Loess smoothed curves and a 95% confidence interval for each smoothing (shaded bands around lines). Each mouse was weighted 5-6 times. Untreated R6/1 HD control mice n = 20, 11 males and 9 females; anthocyanin-treated R6/1 HD mice n = 23, 15 males and 8 females.

Female R6/1 HD mice continue to gain weight throughout the experiment regardless of treatment. However, the growth curves for anthocyanin-treated and untreated R6/1 HD female mice diverge at around 20 weeks of age. As indicated by the 95 % confidence interval around the growth curves in Fig. 1A, anthocyanin-treated female R6/1 HD mice gained significantly less weight than female R6/1 HD untreated mice after this point.

For anthocyanin-treated and untreated R6/1 HD male mice the growth curves begin to diverge at around 11 weeks of age. As indicated by the 95 % confidence interval around the growth curves in Fig. 1B, anthocyanin-treated R6/1 HD male mice were significantly lighter than untreated R6/1 HD male mice up to 21 weeks of age. Notice that both treated and untreated male R6/1 HD mice lost weight from about 14 weeks of age, which is a genotype effect 34 ^,^ 39 ^,^ 40. It appeared that the anthocyanin-treated male R6/1 HD mice maintained their body weight better than untreated R6/1 male mice from about 16 to 22 weeks of age. In contrast, no weight loss was observed in anthocyanin-treated and untreated R6/1 HD female mice.

**CAG repeat expansion in R6/1 mice**

CAG repeat expansion has been shown to be reduced in mice lacking glycosylases that excise oxidative lesions from DNA, such as Ogg1 8 and Neil1 9. Accordingly, we hypothesized that treatment with anthocyanin antioxidants would reduce the number of oxidative lesions that are repaired by these glycosylases, thereby leading to reduced repeat expansion. We investigated whether treatment with anthocyanin antioxidants could reduce CAG repeat expansion in R6/1 mice. A biopsy from the ear was taken from each mouse at 3 weeks of age and used as a reference value for the number of CAG repeats present at this time point. At birth the repeat length in tail and liver are identical 41. Somatic expansion becomes apparent in some organs at six weeks of age (Supplementary Fig. S1). Samples from several organs of R6/1 HD mice were obtained at 22 weeks of age, including ear, kidney, liver, olfactory bulb, cortex, striatum, testis and sperm. The isolated DNA from these samples was analysed for CAG repeat length. Representative examples of GeneMapper raw data from all organs of one anthocyanin-treated and one untreated R6/1 HD mouse at 22 weeks of age are shown in Supplementary Fig. S2. The average CAG repeat lengths in the analysed organs from anthocyanin-treated and untreated R6/1 mice are shown in Supplementary Fig. S3.

A method to parameterize tissue-specific repeat instability has been published by Lee et al. 42. We applied a similar approach to determine the instability index (see Methods). In short, the magnitude of the peak heights included in the fragment analysis need to be normalized to one, multiplied by the distance to the main allele, and then summed to obtain the instability index. The calculated normalized peaks for each organ of anthocyanin-treated and untreated R6/1 HD mice are displayed in Supplementary Fig. S4.

Anthocyanin-treated R6/1 mice had reduced instability index in ear (P < 0.01) and cortex (P < 0.01) compared to untreated R6/1 mice (Fig. 2).

**CAG repeat expansion measured by the instability index.**
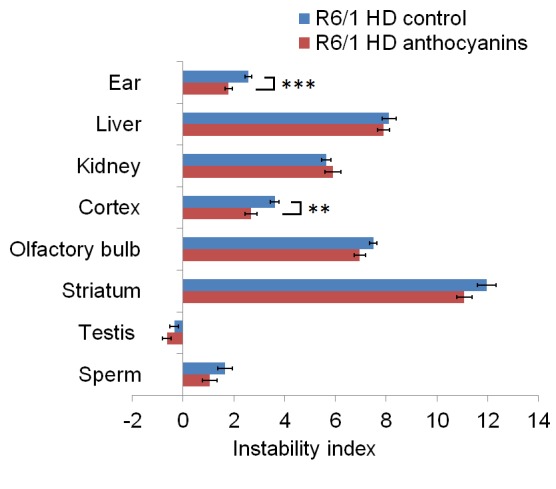
The instability index calculated in several organs of anthocyanin-treated and untreated R6/1 mice. In order to measure the instability index, the magnitude of the peak heights included in the fragment analysis need to be normalized to one to the peak height of the main allele, multiplied by the distance to the main allele, and then summed (see also Methods, Supplementary Fig. S4 and 42). Untreated R6/1 HD control mice n = 19, 10 males and 9 females; anthocyanin-treated R6/1 HD mice n = 23, 15 males and 8 females; data shown as means ± S.E.M.). * P < 0.05; ** P < 0.01; *** P < 0.001; two-tailed unpaired *t*-test.

In striatum and olfactory bulb the P-values for the instability index were 0.07 for both. The negative instability index numbers measured in testis indicated CAG repeat contraction, which was not different between anthocyanin-treated and untreated mice (P = 0.25). No differences in instability index were detected in sperm (P = 0.18), kidney (P = 0.47) and liver (P = 0.55).

**Effects of anthocyanins on R6/1 mice in the open field test**

Spontaneous locomotor activity, as a general measure of motor function and emotional state, was measured in the open field test 43. The measures of primary interest in the open field were the total distance travelled, the entries into, and the percent time spent in the centre square (20 x 20 cm) of the arena.

A two-way analysis of variance (ANOVA) showed no statistically significant difference between anthocyanin-treated and untreated R6/1 HD mice at 22 weeks of age on the total distance travelled during the whole 45 minutes of the test (P = 0.570; Fig. 3A and 3B).

**The open field test of activity, locomotion and anxiety-like behaviour.**
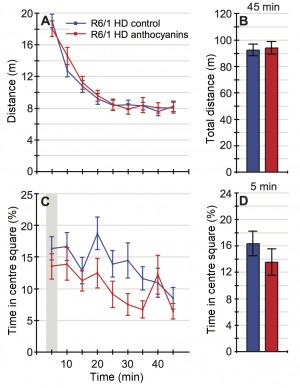
The 22-week old anthocyanin-treated and untreated R6/1 HD mice were placed in an open arena of 40 x 40 cm and their movement were recorded for 45 minutes from a video camera placed above. **A)** and **B)** The total distance travelled showed no significant main effect of anthocyanin treatment. **C** ) Percent time in the centre square (20 x 20 cm) of the open field arena during the whole 45 minutes test and **D** ) during the first 5 minutes of the test. Untreated R6/1 HD mice n = 20, 11 males and 9 females; anthocyanin-treated R6/1 HD mice n = 23, 15 males and 8 females; two way ANOVA; data shown as means ± S.E.M.

Irrespective of treatment, the female R6/1 HD mice travelled significantly further than male R6/1 HD mice (P = 0.004, two-way ANOVA; data not shown). There was no significant interaction between treatment and sex (P = 0.776, two-way ANOVA; data not shown). During the time course of 45 minutes, anthocyanin-treated R6/1 HD mice appeared to spend less time in the centre compared to untreated R6/1 HD mice (Fig. 3C).

As an index of anxiety-like behaviour, we analysed time spent in the central square of the arena during the first 5 minutes of the observation period. During the first five minutes of the test, there was no statistically significant difference in the time spent in the centre between anthocyanin-treated and untreated R6/1 HD mice at 22 weeks of age (Fig. 3D; P = 0.430; two-way ANOVA), indicating no effects of anthocyanins on anxiety-like behaviour. There were no significant sex differences or interactions on the time spent in the centre (two-way ANOVA; data not shown).

In addition, we analysed a subset of 20-week old R6/1 wild-type (WT) mice and compared them to HD siblings in the open field test (Supplementary Fig. S5). The HD mice used here were not the same as the untreated HD mice in Fig. 3. No statistically significant differences were detected between genotypes in the total distance travelled or in anxiety-like behaviour (Supplementary Fig. S5D). During the whole 45 minutes test, R6/1 HD mice appeared to spend more time in the centre compared to R6/1 WT mice, particularly later in the time course (Fig. S5).

**The effect of anthocyanin treatment on the latency to fall in the rotarod test**

Motor coordination and balance performance was measured on an accelerating rotarod 44. A two-way repeated measures ANOVA showed no significant main effect of sex on the latency to fall from the accelerating rotating drum (P = 0.469). Using aggregated data from both sexes, the latency to fall was close to significance for anthocyanin-treatment, (Fig. 4; P = 0.0525). Furthermore, there were no significant interactions between any of the variables.

**Motor coordination of 22 weeks old anthocyanin-treated and untreated R6/1 HD mice on the rotarod test.**
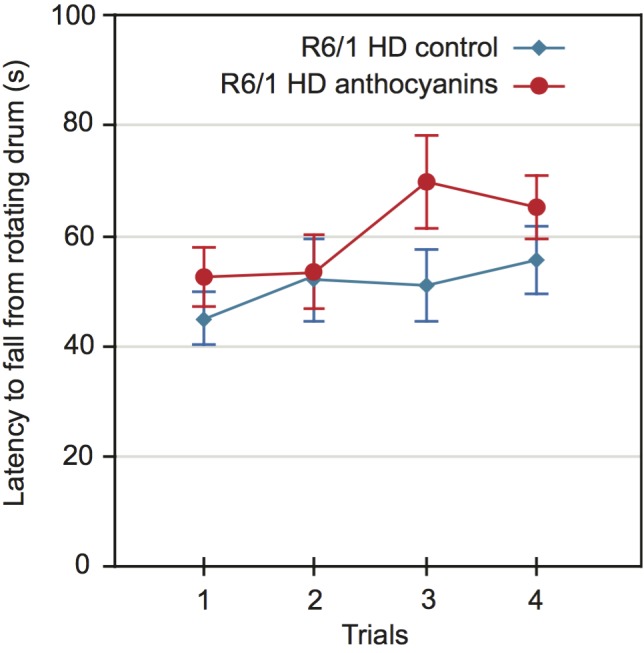
The latency to fall from a rotating drum was repeatedly measured in the accelerating rotarod test of balance and coordination. There was a tendency towards improved latency to fall from the rotating drum after anthocyanin treatment at 22 weeks of age (P = 0.0525, two-way repeated measures ANOVA). Untreated R6/1 HD mice n = 20, 11 males and 9 females; anthocyanin-treated R6/1 HD mice n = 23, 15 males and 8 females; data shown as means ± S.E.M.).

Since anthocyanin-treatment appeared to affect the weight of the mice, we decided to include weight as a parameter in our ANOVA test. Lighter mice performed significantly better than heavier mice on the rotarod (P = 0.0023).

Since mouse weight affected rotarod performance, we correlated mouse weight with the average latency to fall for each mouse individually (Supplementary Fig. S6). There was a negative correlation between weight and rotarod performance, and no significant effect of anthocyanin treatment. There were no gender differences.

## Discussion

**Effects of anthocyanins on body weight**

The growth rate up to about 10 weeks of age was not affected by anthocyanins in either female or male R6/1 HD mice. The growth curve in R6/1 HD female mice in our study was not affected by anthocyanin-treatment until 20 weeks of age. Another study has shown that R6/1 HD female mice reached a maximum body weight at 22 weeks of age and then showed a decline 45. Therefore, it is likely that we terminated the experiment when the R6/1 HD female mice reached their maximum body weight.

From about 11 weeks of age, the growth rate of anthocyanin-treated male R6/1 HD mice was significantly reduced compared to untreated male R6/1 HD mice (Fig. 1B). Male C57BL/6J mice on a high fat diet, given purified anthocyanins from blueberry, have been shown to have lower body weight gains and body fat than controls 46. Anthocyanin consumption has also been shown to lower epididymal fat, blood glucose and cholesterol in rats 47. These data by others may explain why the anthocyanin-treated R6/1 HD mice had less maximal adult body weight compared to untreated R6/1 HD mice.

From about 14 weeks of age, we observed weight loss among male R6/1 HD mice. Male HD R6/1 and HD-N171-82Q transgenic mice show weight loss from about 12-14 weeks of age compared to WT, which is a genotype effect 34 ^,^ 39 ^,^ 40. The weight loss appeared to be more rapid in untreated male R6/1 HD mice (Fig 1B). Likewise, HD-N171-82Q transgenic mice given a combination of coenzyme Q10 and remicade hydrochloride dietary supplements maintained body-weights better than mice on standard diet 34. On the other hand, treatment with essential fatty acids, resveratrol, or *N*-acetylcysteine did not ameliorate weight loss in HD transgenic mice 37 ^,^ 39.

**CAG repeat expansion**

The instability index was reduced in ear and cortex of anthocyanin-treated compared to untreated R6/1 HD mice. When checking for multiple testing with Bonferroni correction (different tissues, n=8), all differences became non-significant, although CAG expansion in different tissues may not be considered as independent tests.

In R6/1 mice, we have previously demonstrated periodic expansions in striatum and cortex brain tissues, and continuous expansion in tail 6. Since large and periodic expansions were observed in both anthocyanin-treated and untreated mice (Supplementary Fig. S7), it seems that anthocyanins have a negligible effect on these types of expansion in brain. However, the continuous expansion in ear was significantly inhibited by anthocyanins, indicating that anthocyanin treatment may reduce expansion in other cell types that display continuous expansion.

One study has shown that the HTT transgene in R6/1 has two CAG repeat tracts 48, which could be relevant for repeat instability analysis. However, the presence of two CAG repeat tracts does not fit with the original analysis 5 and has not been reported elsewhere.

When calculating the instability index, we used the highest peak in the fragment analysis of the ear sample at three weeks of age as the main allele, while Lee et al. 42 apparently used the highest peak of the tail sample at 5 months of age. Although repeat expansion in the tail is considered to be insignificant, we have shown that the CAG repeat length expands with approximately 2 repeats during an 18-week period 6. A main allele from samples taken at five months of age might therefore give a higher starting point when calculating the instability index, leading to false contractions.

Small pool PCR has previously been used to measure repeat instability. GeneMapper traces and instability indices from bulk DNA and frequency distributions of CAG repeat lengths obtained from small pool PCR are highly correlated 42. Although the bulk DNA method may not detect rare large expansion, it gives a good estimate of overall instability.

Overall, anthocyanin-treatment did not affect repeat expansion in R6/1 mice considerably, which indicates that either the antioxidant properties of anthocyanins are insufficient to affect oxidative damage levels and expansion to a major extent, or that other factors than oxidative damage regulate the main expansion processes. The anthocyanin dose used in this study was high. Unfortunately, we were unable to measure the levels of anthocyanins in target tissues. However, anthocyanins have been detected in several brain regions in rats 22 ^,^ 23 and pigs 24 after ingestion of dietary blueberry, demonstrating that anthocyanins penetrate the blood-brain barrier.

**CAG repeat instability in male gonads**

We did not demonstrate any significant effects of anthocyanin-treatment on repeat instability in male gonads of R6/1 mice. The instability index in testis was negative for both treatment groups, indicating CAG repeat contraction compared to the 3-week ear reference. We cannot exclude that CAG repeats slightly expanded from 0 to 3 weeks in the ear. In Supplementary Fig. S3 we have shown that the average CAG repeat length in ear expanded 2.6 repeats from 3 to 22 weeks of age in untreated R6/1 HD mice, giving an estimate of 0.137 repeats per week. This indicates a maximum of 0.41 repeat increases from birth to three weeks of age in the ear if the expansion rate is constant. Kovtun et al. showed that expansions occurred when spermatids developed into spermatozoa 49, which correlates with our finding that the average CAG repeat length in sperm were longer than in testis from untreated R6/1 mice. However, in humans, expansions have also been reported in pre-meiotic and meiotic cells in testis 50. It is well documented that the repeat length may increase from father to child probably because of large instability in the human sperm 51 ^,^ 52 ^,^ 53.

**Behavioural studies**

Anthocyanin-treatment did not affect the total distance travelled in R6/1 HD mice. Similarly, there were no significant differences in the total distance travelled among R6/1 WT and HD mice. However, treatment with *vaccinium* berries has been shown to increase the number of crossings in the open field habituation in adult rats 28 and mice 26.

There were no statistically significant differences between anthocyanin-treated and untreated R6/1 HD mice in the time spent in the centre during the first five minutes of the open field test, indicating no effects of anthocyanins on anxiety-like behaviour in R6/1 HD mice at 22 weeks of age. Likewise, among HD and WT R6/1 mice at 20 weeks of age, no differences in anxiety-like behaviour were observed. R6/1 mice at 12 weeks were also unaltered in anxiety levels 33. In contrast, mice and rat models of HD have previously been shown to spend more time in the open area compared to WT in the elevated plus-maze test, indicating reduced anxiety 54 ^,^ 55.

Later in the time course of the open field test, it appeared that anthocyanin-treated HD mice spent less time in the centre compared to untreated HD mice. R6/1 HD also appeared to spend more time in the centre late in the time course, compared to R6/1 WT mice. This might reflect a cognitive deficit, that the HD mice no longer realize that the centre is a more “dangerous place”. Indeed, R6/1 HD mice show a deficit in short-term spatial memory on the T-maze compared to R6/1 WT mice at 12 weeks of age 33.

HD transgenic mice and rats display a progressive motor deficit on the rotarod 40 ^,^ 55 ^,^ 56 ^,^ 57. The latency to fall in anthocyanin-treated R6/1 HD mice at 22 weeks of age was close to statistical significance. Since body weight affected rotarod performance, we correlated body weight with the latency to fall, and found no difference in treatment. Others have shown that antioxidant treatment improved rotarod performance in HD transgenic mice to some extent 34 ^,^ 36 ^,^ 38 ^,^ 39 ^,^ 57. After testing rotarod performance longitudinally, environmental enrichment delayed the latency to fall in R6/1 HD mice 58. Anthocyanin treatment could have affected rotarod performance earlier in the disease course, but we have no data to confirm this hypothesis.

One study found a beneficial effect on age-related decline in rotarod performance in 19-month old rats given blueberry supplementation 29. We did not test anthocyanin-treatment in R6/1 WT animals and cannot rule out that anthocyanins may affect behaviour in WT mice.

There were more males than females in the R6/1 HD treatment groups. If there are gender differences this could influence the studies. However, the distribution of males and females between each treatment groups were similar. In addition, we did not observe any gender differences within the same treatment group, with the exception of females travelling further in the open field than males, which was not affected by treatment.

**Conclusion**

In conclusion, the instability index was reduced in the ear and the cortex of anthocyanin-treated compared to untreated R6/1 mice. The time spent in the centre square zone during the first five minutes of the open field test was neither significantly different between anthocyanin-treated and untreated R6/1 HD mice, nor between R6/1 HD and R6/1 WT mice. These results indicate no effects on anxiety-like behaviour. During the open field time course of 45 minutes R6/1 HD mice appeared to have cognitive deficits. Anthocyanin treatment may improve this deficit in R6/1 HD mice, although further studies are required to confirm this. Mouse weight affected the latency to fall in the rotarod test. Longitudinally behavioural studies could clarify whether anthocyanins have an effect on the behaviour of R6/1 HD mice earlier in the disease course. More studies of HD mice are warranted to investigate if anthocyanins can delay the onset and progression of HD symptoms.

## Methods

**Ethics statement**

All experimental procedures were approved by the section for comparative medicine at Oslo University Hospital and the Norwegian animal research authority, and complied with national laws, institutional regulations and EU Directive 86/609/EEC governing the use of animals in research.

**Anthocyanin content in Medox^®^ capsules**

The anthocyanins were obtained from opened Medox^®^ capsules (MedPalett Pharmaceuticals AS, No) and consisted of purified anthocyanins isolated from bilberries *(Vaccinium* *myrtillus)* and blackcurrant *(Ribes* *nigrum).* The powder consists of a mixture of 3-*O*-rutinosides of cyanidin and delphinidin, and 3-*O-β*-galactosides, 3-*O-β*-glucosides, 3-*O-β*-arabinosides of cyanidin, peonidin, delphinidin, petunidin, and malvidin. The 3-*O-β*-glucosides of cyanidin and delphinidin constituted about 40-50% of the total anthocyanins.

**Animals and treatment with anthocyanins**

Two male B6CBA-Tg(HDexon1)61pb/J mice of the R6/1 line 5 from the same litter with ~115 CAG repeats in exon 1 of the human *HTT* gene were crossed with C57BL/6 female mice, and the offspring carrying the human *HTT* fragment were divided into two groups; the anthocyanin-treated group (23 mice) and the control group (20 mice). The female to male ratio was 9:11 for the control group, and 8:15 for the treatment group. The anthocyanin-treated group was given Medox^®^ stirred in filtered tap water (1,6 g/L), freshly made every day and given from the age of 3-4 weeks. The control group received only plain filtered tap water. The R6/1 mice were weighed regularly and intake of drinking water was measured, giving an estimate of daily anthocyanin intake of approximately 300 mg/kg bodyweight/day. There were no significant differences in water intake between the groups (data not shown). The R6/1 mice were housed in transparent polycarbonate cages with controlled temperature and humidity, and fed Rat and Mouse No.1 maintenance diet (Special Diet Services) and drinking water *a* *d libi* *tum.* R6/1 WT and HD mice were subjected to behavioural tests at 20 or 22 weeks of age, respectively. Testing was conducted during the light phase (0600-1800 h) of the light/dark cycle. The R6/1 mice were brought to the laboratory at least 30 minutes before testing, and the open field test was performed the day before the rotarod test.

**Tissues, genotyping and sizing of CAG repeats**

At 3 weeks of age an ear biopsy was taken from each mouse for genotyping and to measure the number of CAG repeats present at this age. At 22 weeks of age the mice were sacrificed by cervical dislocation, the organs were harvested, frozen on dry ice and stored at −70°C. Sperm was collected by excising the epididymis from the testis, then squeezed out and washed two times in PBS by centrifugation at 400g for 3 min. DNA from all tissues was isolated according to the DNeasy^®^ Blood & Tissue kit (Qiagen GmbH, Germany). The kit procedure was modified for sperm DNA by following protocol 2 on the Qiagen website. CAG repeats were sized by PCR with primers 5’-FAM-atgaaggccttcgagtccctcaagtccttc-3’ and 5’-ggcggctgaggaagctgagga-3’ according to 5 with slight modifications. Approximately 75ng of Genomic DNA was amplified with 0,15U AmpliTaq^®^ Gold DNA polymerase (Applied Biosystems, CA), PCR Buffer II, 1.25 mM MgCl2, and 2.5 mM dNTPs (Applied Biosystems). The cycling conditions were 94°C for 10 min, 35 cycles of 94°C for 30 sec, 64°C for 30 sec, 72°C for 2 min, and a final extension at 72°C for 10 min. The FAM-labeled PCR products were mixed with GeneScan™ - 600 LIZ^®^ Size Standard and HiDi™ Formamide (Applied Biosystems) and run on an ABI 3730 Genetic Analyzer (Applied Biosystems). Sizing of the PCR fragments was done by using the GeneMapper^®^ Software Version 3.7 (Applied Biosystems). Levels of anthocyanins in different tissues were not measured.

**Calculation of instability index**

Calculation of instability index was done with a custom script in MATLAB (R2012b; MathWorks Inc., USA) as previously described 42, with minor changes: We used a 10 % peak threshold of the highest peak from the GeneMapper sample plots instead of 20 %, and ear samples taken at 3 weeks of age instead of tail samples. Ear and tail samples from the same mouse have shown identical CAG repeat values at 3 weeks of age (Møllersen et al., unpublished results). Normalized peak heights were calculated by dividing the peak height of each peak by the sum of all peak heights. The change in CAG repeat length was determined by referring to the main allele (the highest peak in the ear sample of the mouse at three weeks of age), and then giving the peaks in the sample from 22 weeks a position number according to their distance from the main allele. The normalized peak heights were then weighted by multiplication with the position numbers, and the instability index was the sum of the normalized and weighted peaks.

**Open field test**

The open field test was conducted in four identical square arenas (L40 cm x W40 cm x H35 cm), custom made from white PVC plastic. The arenas were evenly and indirectly illuminated from above (~200 lux). The R6/1 mice were individually placed along a wall of the open field and allowed to explore freely for 45 minutes. Spontaneous exploratory activity was monitored by a ceiling-mounted camera (Creative NX Ultra, Creative Technology Limited, Singapore) and tracked in real time by a video tracking system (ANY-maze, Stoelting Co., USA). The experimenter left the room after initiating the open field test and returned at the end of the 45 minutes test.

**Rotarod test**

The rotarod test was conducted on a TSE RotaRod *Advanced* with an Ø = 30 mm grooved drum for mice and a fall height of 15.8 cm (TSE Systems GmbH, Germany). The R6/1 mice were placed on the drum in one of four lanes facing away from the experimenter. Three 60 sec practice trials, with the drum rotating at 4 rpm, were given with an inter-trial interval of 10 minutes. Thirty minutes after the last practice trial the first of four test trials were given. During the test trials the drum rotation accelerated from 4 to 40 rpm over 300 seconds. The inter trial interval between test trials was fifteen minutes. The latency to fall was taken as an indicator of motor coordination and balance.

**Statistical analysis**

Student’s *t*-test was used for normally distributed data to compare average CAG repeat lengths and the instability index in all sampled organs between the anthocyanin-treated and the untreated *HTT* transgenic mice (Excel and MATLAB). Student’s *t*-test, Mann-Whitney test, two-way ANOVA, and repeated measures ANOVA (rmANOVA) were used as appropriate to analyse the effects of treatment, sex, weight and trials on the behavioural tests (Minitab, R and ANY-maze). A P-value < 0.05 was considered significant.

## Abbreviations

8-oxoG, 7,8-dihydroxy-8-oxoguanine; BER, base excision repair; HD, Huntington’s disease; HTT/Htt, Huntingtin; Neil1, nei endonuclease VIII-like 1 (E. coli); Ogg1, 8-oxoguanine DNA glycosylase; WT, wild-type.

## APPENDIX

**Supplementary figures**

**Figure:**
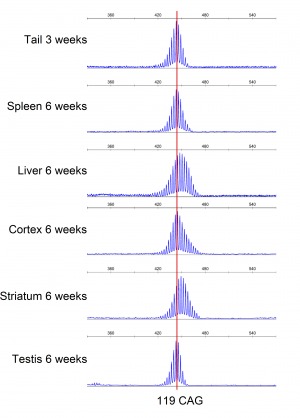
**Supplementary Fig. S1:** GeneMapper raw data from one male R6/1 HD mouse at six weeks of age. The repeat length in tail at three weeks is identical to spleen and testis at six weeks, and only slightly increased in liver, cortex and striatum. These data indicate that there is no repeat instability in spleen and testis, and minimal expansion in liver and brain, from three to six weeks of age in R6/1 mice. The red line indicate the mean number of CAG repeats present in tail at 3 weeks.

**Figure:**
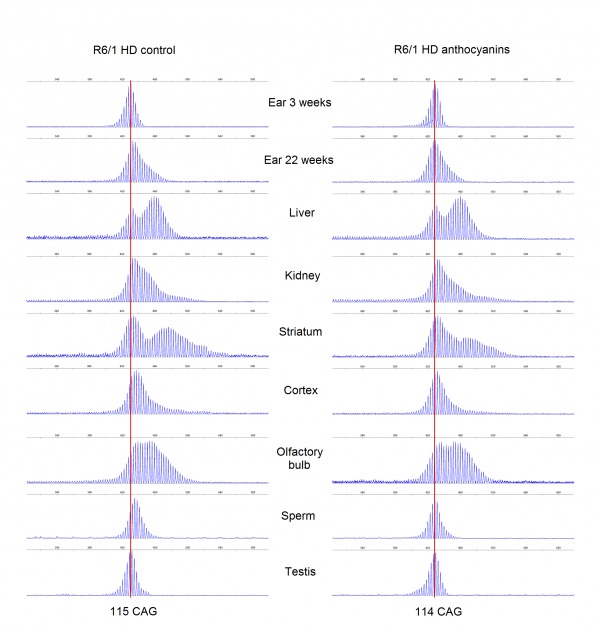
**Supplementary Fig S2:** GeneMapper traces. Displayed plots from GeneMapper showing the fragment distribution in the organs of one male anthocyanin-treated R6/1 mouse and one male R6/1 control mouse at 22 weeks of age. The red lines indicate the mean number for CAG repeats present in ear at 3 weeks.

**Figure:**
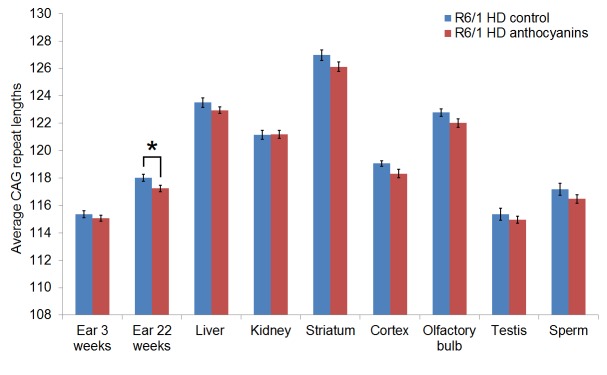
**Supplementary Fig. S3:** The average CAG repeat lengths in several organs from anthocyanin-treated and untreated R6/1 HD mice populations. The mean CAG repeat length from the organs of each mouse was calculated from the GeneMapper raw data using the formula ((∑(peak heights x basepair lengths)/∑peak heights)-86)/3 with a 10% peak threshold. Untreated R6/1 HD control mice n = 20, 11 males and 9 females; anthocyanin-treated R6/1 HD mice n = 23, 15 males and 8 females; data shown as means ± S.E.M.). * P < 0.05; two-tailed unpaired *t*-test.

**Figure:**
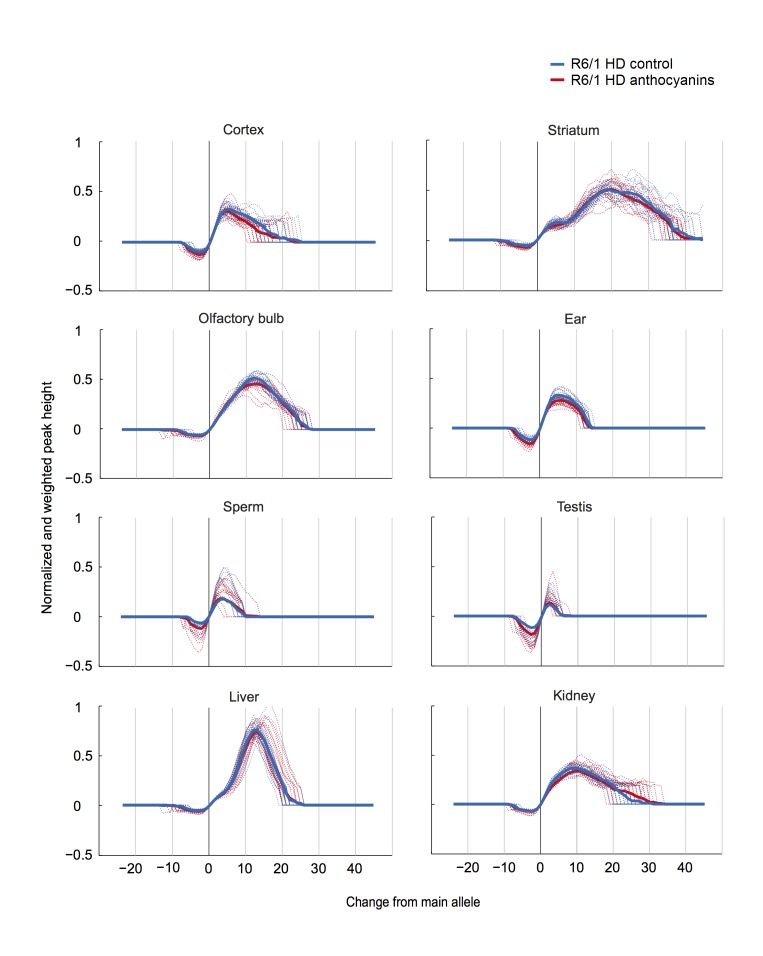
**Supplementary Fig. S4:** Normalized and weighted peak heights for every organ in each R6/1 HD mouse. Dotted lines show the normalized and weighted peak heights for one mouse, while the thick lines display the mean values for the treatment groups.

**Figure:**
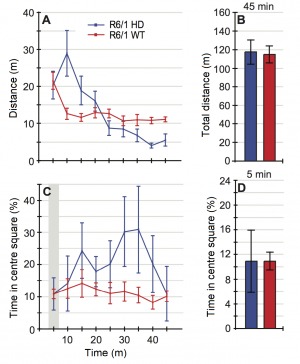
**Supplementary Fig. S5:** R6/1 WT and HD mice of 20 weeks of age were tested in the open field test. **A)** and **B)** There was no difference in the total distance travelled among genotypes during the 45 minutes test (P = 0.519 Mann-Whitney). However, it appeared that the R6/1 HD mice had higher activity in the beginning of the test that declined over time, while the R6/1 WT mice had more stable activity after the first 5 minutes. **C)** After the initial time period, R6/1 HD mice appeared to spend more time in the centre overall, while the R6/1 WT mice continued to avoid spending time in the centre arena. **D)** During the first five minutes of the test, there were no significant differences between R6/1 WT and HD siblings in the time spent in the centre of the arena (P = 0.606, Mann-Whitney). Because the sample sizes were small in this experiment, no meaningful analysis could be performed with sex as an additional factor. Data is shown as means ± S.E.M; n = 8 WT (5 females, 3 males), and n = 6 HD (3 females and 3 males).

**Figure:**
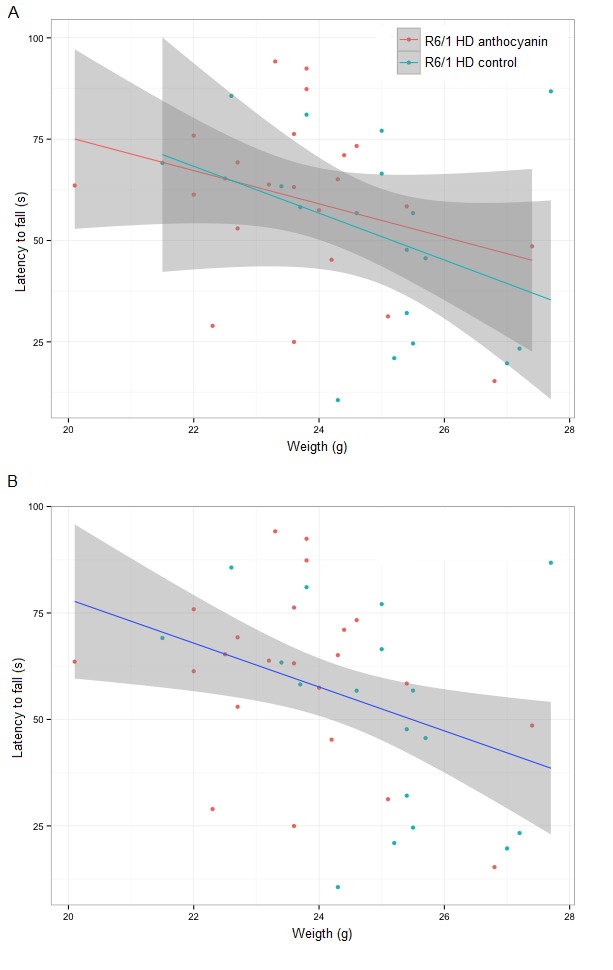
**Supplementary Fig. S6:** Correlation of the mouse weight with the average latency to fall from the rotarod. **A)** Trend lines for anthocyanin-treated and untreated R6/1 HD mice show both a negative correlation. The 95 % confidence intervals for each treatment groups shown in grey are overlapping and indistinguishable, indicating no significant difference in treatment. **B)** From a linear model with combined treatment groups the latency to fall equals 181.24 – 5.15*weight. The coefficient for weight has a P-value of 0.014, confirming that mouse weight affects the latency to fall. Untreated R6/1 HD control mice n = 18, 11 males and 7 females; anthocyanin-treated R6/1 HD mice n = 23, 15 males and 8 females.

**Figure:**
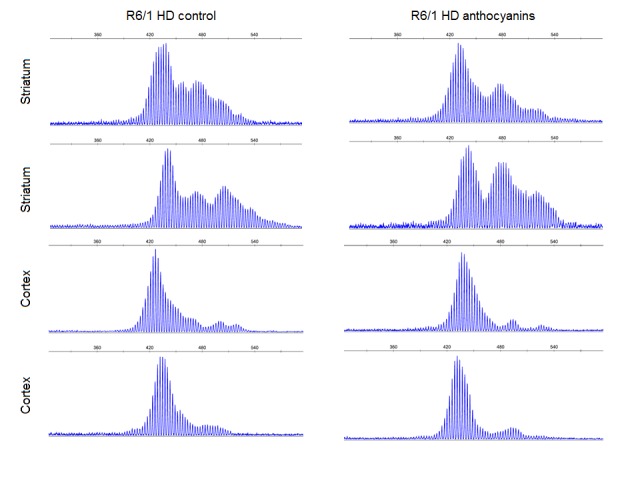
**Supplementary Fig. S7:** Examples of striatum and cortex samples from anthocyanin-treated and untreated R6/1 mice HD showing periodicity as previously reported 6. Most of the striatum samples and up to about half of the cortex samples displayed clear periodicity. No apparent differences were seen in periodicity between anthocyanin-treated and untreated R6/1 HD mice. Periodicity was not observed in any other organs than striatum and cortex.
